# Proceedings of Central Illinois Medical Society

**Published:** 1873-02-15

**Authors:** J. Wright, W. G. Cochran

**Affiliations:** President; Bloomington; Secretary; Bloomington


### PROCEEDINGS OF CENTRAL ILLINOIS MEDICAL SOCIETY.

BLOOMINGTON, Jan. 14, 1873.

The Central Illinois Medical Society met in regular session, Dr. J. Wright, President, in the Chair.

Reading minutes of last meeting postponed till afternoon session. Regular order of business suspended, and on recommendation of Dr. R. G. Laughlin, the Society proceeded to elect the following members :

Drs. T. F. Worrell, J. L. White, D. L. Crist, A. P. Tenny, H. C. Crist, D. O. Moore, H. C. Luce, and N. B. Cole, all of Bloomington; and W. L. Pollock, M.D., of Heyworth.

On motion, adjourned to meet at two o'clock P.M.

AFTERNOON SESSION.

Called to order by President Wright.

Minutes of previous meeting read and approved.

On recommendation of Dr. Laughlin, J. Little, of Le Roy, and L. Asire, of Bloomington, were elected members of the Society.

Dr. J. J. Starkey, of the Louisville Medical College, was invited to participate in the proceedings of the Society.

Dr. W. Hill, of Committee on Surgery, made a valuable and very interesting report on Lythotomy, criticising severely some of the modern modes of operating.

On recommendation of Dr. Tenney, Dr. Lee Smith, of Rush Medical College, was elected a member of the Society.

Dr. S. H. Birney, Chairman of Committee on Obstetrics and Diseases of Females, read a very interesting essay on Diseases of Females; the production was a valuable one, and did great credit to its author.

On motion, Dr. Hill and Dr. Birney were requested to furnish copies of their essays for publication in the Chicago Medical journals.

On motion, the publishing Committee were instructed to have one hundred copies of the Constitution and By-laws printed.

*Delegates to State Medical Society:*—S. H. Birney, W. G. Cochran, C. T. Orner, and W. L. Pollock.

*Delegates to American Medical Society:*—H. C. Luce, C. Goodbrake, S. H. Birney, J. Little, G. W. Barton, Jno. Clouser, and W. Hill.

On motion, adjourned to meet at seven o'clock P.M.

EVENING SESSION.

Called to order by Dr. Wright, President.

Dr. Laughlin, of Committee on Practical Medicine, made a practical and very interesting report on the diseases peculiar to this country since the last meeting of the Society. The report elicited a lively discussion, and the Doctor was requested to furnish a copy of it for publication in the Chicago Medical journals.

Dr. J. S. White, of Bloomington, delivered a very interesting address. Although prepared for the public, it was very interesting to the Society, as was manifested by a resolution of thanks to the Doctor for his valuable production.

The President announced the following Special committees :

*Surgery:*—Drs. H. C. Luce, C. Goodbrake, and S. H. Birney.

*Practical Medicine:*—Drs. J. T. Pearman, Z. H. Madden, and G. W. Barton.

*Obstetrics and Diseases of Females:*—Drs. M. S. Brown, A. P. Tenney, and J. H. Potter.

*Pathology:*—Dr. C. T. Orner.

*Microscopy:*—Dr. R. D. Bradley.

*Eye and Ear:*—Drs. N. B. Cole, H. C. Howard, and R. H. Huddleston.

*Committee of Arrangements:*—Drs. S. H. Birney, J. T. Pearman, and H. C. Howard.

Very interesting verbal reports were made by Drs. Worrell, Orner, and others.

The following resolution was unanimously adopted :

*Resolved*, That the thanks of the Society are due to the Committee of Arrangements, the physicians of the city, the authorities of the county, and trustees of the Methodist church for kind care and hospitalities shown us while within their midst.

On motion, the Secretary was instructed to furnish copies of proceedings for publication in the county papers.

On motion, adjourned to meet in Champaign, the second Tuesday in June, 1873.

Notwithstanding this was the first meeting of the Society since its organization, it was well attended, and the greatest interest was manifested. We expect, however, to have a still more interesting time in Champaign, next June, where we hope to meet all the physicians of the central portion of the State, and *every one* surcharged with "medical news." J. WRIGHT, *President.*

W. G. COCHRAN, *Secretary.*

DEATH OF A NOTED SOUTHERN SURGEON.—Dr. Warren Stone, long a distinguished surgeon of New Orleans, Louisiana, died in that city, December 6, 1872, aged 65 years.

